# Inter-Level Feature Balanced Fusion Network for Street Scene Segmentation

**DOI:** 10.3390/s21237844

**Published:** 2021-11-25

**Authors:** Dongqian Li, Cien Fan, Lian Zou, Qi Zuo, Hao Jiang, Yifeng Liu

**Affiliations:** 1 School of Electronic Information, Wuhan University, Wuhan 430072, China; 2015301220070@whu.edu.cn (D.L.); fce@whu.edu.cn (C.F.); 2019202120073@whu.edu.cn (Q.Z.); jh@whu.edu.cn (H.J.); 2 National Engineering Laboratory for Risk Perception and Prevention (NEL-RPP), Beijing 100041, China; liuyifeng3@cetc.com.cn

**Keywords:** semantic segmentation, encoder–decoder, feature balanced fusion, Cityscapes

## Abstract

Semantic segmentation, as a pixel-level recognition task, has been widely used in a variety of practical scenes. Most of the existing methods try to improve the performance of the network by fusing the information of high and low layers. This kind of simple concatenation or element-wise addition will lead to the problem of unbalanced fusion and low utilization of inter-level features. To solve this problem, we propose the Inter-Level Feature Balanced Fusion Network (IFBFNet) to guide the inter-level feature fusion towards a more balanced and effective direction. Our overall network architecture is based on the encoder–decoder architecture. In the encoder, we use a relatively deep convolution network to extract rich semantic information. In the decoder, skip-connections are added to connect and fuse low-level spatial features to restore a clearer boundary expression gradually. We add an inter-level feature balanced fusion module to each skip connection. Additionally, to better capture the boundary information, we added a shallower spatial information stream to supplement more spatial information details. Experiments have proved the effectiveness of our module. Our IFBFNet achieved a competitive performance on the Cityscapes dataset with only finely annotated data used for training and has been greatly improved on the baseline network.

## 1. Introduction

Semantic segmentation is a task to predict the corresponding category of each pixel in the image. This topic is a very popular direction in computer vision and can be applied to many practical tasks, such as city scenes [1,2,3,4,5], satellite topographic survey [6,7], and medical image analysis [8,9,10]. The new point-to-point network structure designed by Fully Convolutional Networks (FCN) for semantic segmentation [11] performs well in this dense pixel prediction task. This structure foundation accelerates the development of semantic segmentation. So far, many excellent networks have been developed [12,13,14,15].

In FCN [11], the multilayer convolution and pooling structure results in a 32-fold reduction in the final feature compared to the input image. This design loses a lot of spatial information, resulting in inaccurate predictions, especially on the edge details of the picture. To solve this problem, many networks have tried various methods. For example, the atrous convolution applied in DeepLabV3 [16] increases the field of perception while not reducing the size of the feature map. There is also a parallel atrous convolution (Atrous Spatial Pyramid Pooling, ASPP) [2,16] structure that can be used to improve the result of the segmentation if added to most of the segmentation networks. Additionally, the encoder–decoder [1,8] network structure is often a countermeasure to the above loss of spatial structure information. In an encoder–decoder network, the backbone network of the classification network is often used as the encoder [11,13,17], which is responsible for encoding the input pictures into feature mappings with low resolution but rich semantic information. The decoder then restores the pixels of the low-resolution feature to obtain a pixel-level category prediction of the same size as the original image, usually designed as a series of convolution and up-sampling operations. Because the direct up-sampling of low-resolution feature maps still lacks the spatial detailed information lost in the encoder, decoders often incorporate low-level features into the up-sampling parts to capture the fine-grained information. A typical structure design is DeepLabV3plus [2].

**Figure 1:**
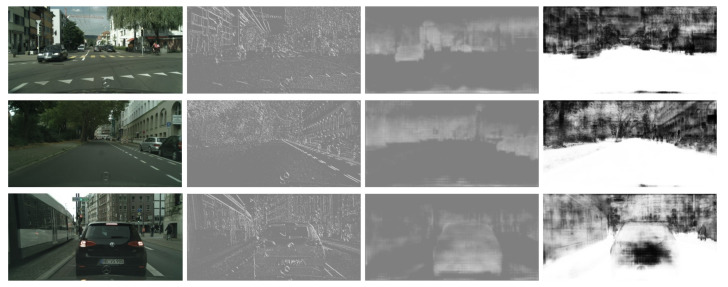
Visualization examples of features in different stages of the concatenation process on the Cityscapes dataset [19]. From left to right are input images, low-level features, deep-level features, and features after concatenation fusion.

Carefully analyzing most of the existing segmentation networks, it is not difficult to find that there are usually two ways to integrate low-level features in decoders: concatenation and element-wise addition. Element-wise addition adds features with the same size and number of channels. When convoluting, a priori is added. By the way of addition, new features can be obtained. This new feature can reflect some unique information of the original features, but some information contained in the original features will be lost in the process. Concatenation is the splicing of feature mappings of the same size in the channel dimension. After splicing, each channel corresponds to the corresponding convolution kernel. Compared with concatenation, addition saves more computation than concatenation, but there is no information loss in the concatenation process without considering the amount of computation. Therefore, to obtain a better prediction result, the concatenation fusion feature is more commonly used in semantic segmentation [2,8,18]. However, this also leads to some problems. Because decoders usually combine in-depth features with shallow features, we know that these two features carry very different types of information. Convolution-output features after direct concatenation are not well integrated, resulting in low information utilization. As shown in Figure 1, the lower parts contain more simple spatial and line information, while the higher features include rich semantic information. The convolution output after concatenation shows that the spatial information in the semantic features has been optimized greatly. However, the overall features are still mixed, resulting in a situation where both the semantic and spatial characteristics are not prominent. This would undoubtedly result in inaccurate fuzzy predictions for pixel-level segmentation tasks.

Inspired by the above work, we designed an inter-level feature balanced fusion module to fuse the inter-level features in a more balanced and efficient manner, which solves the problem of inefficient feature utilization and weak purpose of the regular inter-level feature fusion method of concatenation or element-wise addition. The core idea of this module is inspired by the differences between spatial and semantic features. In the process of two-level feature fusion, the correlation between two-level features is calculated in the channel dimension after concatenation of the channel dimension. The spatial weights of the spatial feature in the channel dimension and the semantic weights of the semantic feature in the channel dimension are given respectively by using the method of a normalized score. Additionally, in the back propagation [20], we continuously update and optimize the weight information parameters. Compared with the previous feature fusion methods, we consider that the information between the fusion features is different. Our feature balanced fusion module design can let the network learn how to integrate the features, guide the two different information interaction balances, each taking its role, and finally contribute to better segmentation prediction.

Overall, our network is based on the encoder–decoder structure, which uses a backbone with a deep convolution feature extraction network to extract rich semantic information, followed by an Atrous Spatial Pyramid Pooling (ASPP) [16] structure to extract multi-scale features to obtain rich contextual information without reducing the feature resolution. The decoder is designed with the skip-connections structure, which restores the high-level feature resolution while fusing with the low-level feature, adds spatial information to the semantic information, and gradually restores the segmentation boundary. To obtain more spatial information, we also designed a shallow branch of spatial flow and fused it with the previous level features. In each level of fusion structure, we applied the feature balanced fusion module to balance the fusion of features from different parts.

Our contributions can be summarized as follows:

1. An inter-level feature balanced fusion module was designed to solve the problem of feature imbalance caused by traditional concatenation or element-wise addition, which makes the fusion more balanced and utilization of features more effective.
2. A shallow spatial stream with only three convolution layers was designed and added into the network, which is fused with the main semantic features before outputting the prediction in the decoder. This further enriches the spatial information.
3. Our IFBFNet achieved a comparative performance of 81.2% to mIoU on the Cityscapes dataset with only finely annotated data used for training, significantly improving over baselines.

## 2. Related Work

### 2.1. Semantic Segmentation

Before FCN [11] is introduced, the CNN convolution layer is connected by several fully connected layers, while FCN replaces the fully connected layers of the network with common convolution layers, finally outputting a feature mapping of the same size as the input. Since FCN proposes such a point-to-point full convolution network to complete the task of semantic segmentation, this has triggered a wave of research on the direction of semantic segmentation. Researchers have been committed to improving the accuracy of pixel-level prediction. Directions can be roughly divided into three groups: a pyramid module, an encoder–decoder structure, and an attention mechanism.

### 2.2. Pyramid Module

For the pixel-level prediction task of semantic segmentation, the segmentation of small objects is in a very awkward situation in the whole image segmentation, which often causes segmentation errors or rough segmentation contours, or is even completely ignored. To solve the problem of small object segmentation, a multi-scale pyramid module has become the main solution, which consists of multi-scale pooling [17,21] or the dilated convolution of different rates [14,16,22]. To obtain a good segmentation prediction, our goal is to minimize the overall stride of the network to prevent feature mapping from becoming too small and losing too much spatial information. However, the reduction of the stride will result in a significant reduction in the final feature receptive field [23,24,25]. The above two problems seemed to be contradictory until the advent of ASPP [16]. It solves this problem by expanding the feature receptive field without sacrificing the spatial resolution of the feature. The given input features are sampled in parallel with the atrous convolution at different dilated rates by ASPP, which is equivalent to capturing the context of an image at multiple scales. In our network, ASPP was chosen for multi-scale feature extraction, because it can ensure that high-level semantic features maintain a receptive enough field without losing too much spatial information at the same time.

### 2.3. Encoder–Decoder

The encoder–decoder network architecture has achieved excellent results in semantic segmentation tasks [1,8,15,26], such as segmentation of urban landscapes [2,17] and medical images [8,9,10]. At present, most of the popular segmentation networks with good performance are based on this framework to modify or add some modules. It uses an encoder to learn more rich and dense semantic features, and then uses a decoder to incrementally increase the resolution of features to achieve segmented output. Meanwhile, low-level spatial features are incorporated into the decoder process to supplement the spatial information lost by the bilinear interpolation up-sampling of high-level features. The encoder typically employs backbone networks, such as ResNet and VGG series [27,28], that are commonly used for image classification [17,21,25], because they have strong feature extraction capabilities, they are well-suited for semantically segmented tasks that require rich deep features. Therefore, the improvement of the performance of a segmentation network of the encoder–decoder architecture mainly depends on the structure design of the decoder and the connection mode design between the encoder and the decoder. If the design is ingenious, it is easy to improve the segmentation score. For example, in the design of DeepLabV3plus [2], the encoder features are upsampled, then concatenated with the low-level features, and finally, the final output is obtained by upsampling the concatenated features. This design greatly improves network performance. AResU-Net [29] uses the network design of UNet as a reference. The features obtained by the encoder are concatenated with the features of the upper level after being sampled at each level, and then the upper sampling is concatenated. In this way, repeated operations are carried out and finally, the output containing multi-level information is obtained. Inspired by these works, our network also adds skip-connections [1,8,11,30,31] to produce clearer boundaries.

### 2.4. Attention Mechanism

Attention mechanisms are designed to mimic the human visual system, selectively focusing on more significant areas rather than dealing equally with the entire scene. Attention not only tells us where the focus is, but also enhances the representation of interests. Our goal is to improve performance by using attention mechanisms: focusing on important features and suppressing unnecessary ones. A lot of work has used this idea to accomplish various computer vision tasks. A compact module was introduced in the excellent network SENet [32] of image classification, which calculates the attention weight of the feature channel by compressing the excitation feature mapping. In SSA-CNN [33], the target detection box is used as a segmentation ground truth and is further used for learning segmentation features. As the attention map of the detection feature, the feature will be fused with the detection feature for detection. OCNet [34] and DANet [35] use a self-attention mechanism to explore the context. In the segmentation network proposed by Chen et al. [36], different scale features are automatically fused according to the weights calculated by constructing an attention model.

Inspired by these efforts, we designed a feature balanced fusion module to learn the convergent attention weights between different levels of semantic segmentation and to guide them towards more efficient fusion rather than a concatenation of simple channel dimensions. The comprehensive experimental results show that this strategy does make the fusion of deep semantic features and shallow spatial features more balanced.

## 3. Approach

**Figure 2:**
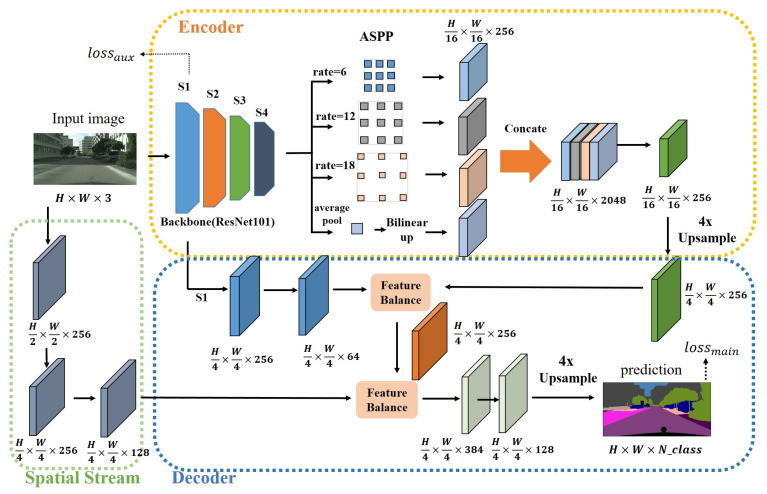
The overall structure of IFBFNet. There are three parts: encoder, decoder, and spatial stream. The encoder is composed of a backbone network and ASPP to extract rich high-level semantic information. In the decoder, we added inter-level feature balanced fusion module into each skip-connection structure. The spatial stream supplements more low-level spatial information.

In this section, we will introduce the details of our network structure design, which is divided into encoder–decoder, feature balance, and shape stream. Our network is mainly composed of three parts: encoder, decoder, and spatial stream. The overall framework of the network is shown in Figure 2.

### 3.1. Our Encoder–Decoder

Based on previous encoder–decoder networks [1,8,15,26], our network also uses the encoder–decoder network architecture. We use ResNet101 [27] as an encoder, which has a strong feature extraction capability, a CNN commonly used for image classification. As shown in Figure 2, We usually divide ResNet into four stages. S1, S2, S3 and S4 in the figure represent stage1, stage2, stage3 and stage4, respectively. The traditional ResNet101 extracts features from the input pictures, and finally reduces the resolution of the feature map to 1/32 of the original input size. This large resolution reduction is negative for the task of outputting a pixel-level prediction of the same size as the input image (H × W × 3). To maintain the resolution of the feature map extracted from the backbone network, we added atrous convolution in ResNet. Specifically, we set dilations as (1,2) and stride sizes as (2,1) in the last two stages of ResNet to obtain the feature map (H/16 × W/16 × 2048) with 1/16 the size of the input image. To obtain context information at multiple scales, we reduced the feature channels extracted by the backbone from 2048 to 256 (H/16 × W/16 × 256) to reduce subsequent computations of ASPP after that, as shown in Figure 2.

The decoder consists of several skip-connections and an upsampling structure designed to restore spatial characteristics. After the process of ResNet and ASPP, we upsample the feature (H/16 × W/16 × 256) four times and concatenate it with the first low-level feature of the backbone. We call the first low-level layer stage one, as shown in S1 (H/4 × W/4 × 256) in Figure 2. To optimize spatial detail information on the premise of preserving most of the semantic information, we use convolution with a kernel size of 3×3, padding of 1×1, and stride of 1×1 to reduce the number of feature channels of stage one (S1) to 64 before concatenation. After the concatenation of the two levels feature, we reduce the channel dimension to 256 (H/4 × W/4 × 256) using two 3×3 convolutions. The feature is then inputted into an inter-level feature balanced fusion module to optimize the feature expression. To complement the spatial feature of S1 to make the prediction map boundary clearer, we add a spatial flow branch to concatenate with the feature balanced above, and then change the channel number of concatenated feature to 256 using 1×1 convolution. After that, the feature map is processed by an inter-level feature balanced fusion module to obtain the optimized features. Finally, we use two 1×1 convolutions to reduce the feature channel dimension from 384 to 128, and then to the number of categories, and then upsample the feature map four times to obtain a prediction with the same size as the original input image (H × W × N_class).

### 3.2. Inter-Level Feature Balanced Fusion Module

In many network architectures ([2,8,18,37]), it is not difficult to find that in the process of restoring the boundary of prediction, the common method is to concatenate the low-level features with high-levels in the channel dimension. For two inputs *X* and *Y*, their sizes are the same, and the numbers of the channel are $C_{1}$ and $C_{2}$, respectively. We assume that the two inputs are $X_{1}$, $X_{1}$, ..., $X_{C1}$ and $Y_{1}$, $Y_{2}$, ..., $Y_{C2}$. Due to the feature concatenation of channel dimensions, differently from the simple feature addition, the subsequent convolution calculation is calculated separately for each channel. Processed by convolution $K_{1}$, $K_{2}$, $K_{3}$, ...,$K_{C1+C2}$, the concatenated feature map $Z_{concat}$’s calculation can be expressed as follows:

(1) $$Z_{concat}=\sum_{i=1}^{c_{1}}X_{i}*K_{i}+\sum_{i=1}^{c_{2}}Y_{i}*K_{c_{1}+i}.$$

where * represents convolution, and $K_{i}$ stands for the ith convolution layer. However, this simple concatenation of high-level and middle-level features of channel dimension results in subsequent features simply as a result of splicing each channel. To some degree, this will ignore the differences between different levels of features and their contribution to the output. As a result, neither of the two levels’ features play their best role. For example, the high-level information contains complex semantic information, while the low-level features mainly represent the features of picture shape, line, color, and texture information. The result is that the simple concatenation cannot express the shallow information well, nor can it express the deep information well.

Inspired by the above, we propose a feature balanced fusion strategy that can guide the two-level features’ fusion towards a more balanced direction and improve the utilization of features. Unlike previous methods, after concatenating the two feature levels $Feat_{h}\in R^{C_{h}\times H\times W}$ and $Feat_{l}\in R^{C_{l}\times H\times W}$, we use an average pooling operation to compress the spatial information of the fused features into a one-dimensional concentrated expression $W\in R^{(C_{h}+C_{l})\times 1\times 1}$, and then use 1×1 convolution to calculate the correlation information between the two features, and finally use sigmoid normalization to obtain the balance weights $W_{h}\in R^{C_{h}\times 1\times 1}$ and $W_{l}\in R^{C_{l}\times 1\times 1}$ of the two-feature fusion. The calculation equation can be seen in (2):

(2) $$W=W_{h}{∥}_{c}W_{l}=Sigmoid(Avgpool\left(Feat_{h}{∥}_{c}Feat_{l})\right).$$

In Equations (2) and (3), ${\left|\right|}_{c}$ represents the splicing of channel dimensions. Then, the weights are multiplied with the original two levels of features to obtain the fused feature map after optimizing the weights, as we describe in Equation (3):

(3) $$Z_{balanced}=W{(Feat_{h}{∥}_{c}Feat_{l})=\left(W_{h}\times Feat_{h}\right)∥}_{c}\left(W_{l}\times Feat_{l}\right).$$

At the same time, in order to preserve some of the primitive information, we added a residual structure at the end, adding the unprocessed features to the optimized feature by element-wise addition.

**Figure 3:**
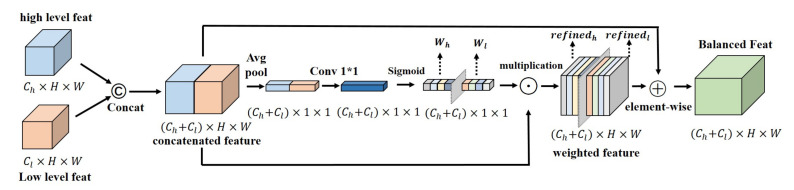
The structure design of the inter-level feature balanced fusion module. *©*, ⊙, and ⨁ respectively stand for concatenation, multiplication, and element-wise addition. The fusion weights of high-level features $W_{h}$ and low-level $W_{l}$ features are calculated respectively.

The specific structure of the inter-level feature balanced fusion module is shown in Figure 3. Overall, after concatenating features at different levels in the channel dimension, we compress the feature map space into 1×1 dimensions and normalize the relationship between the two levels of features to generate the balanced weights of the two levels of features. The formula for calculating the feature after the balance of features is:

(4) $$F_{balanced}=\sum_{i=1}^{c_{h}}W_{hi}Feat_{hi}*K_{i}+\sum_{i=1}^{c_{l}}W_{li}Feat_{li}*K_{c_{h}+i}.$$

By comparing the feature expressions calculated by Equations (1) and (4), it is clear that our feature balanced fusion method yields a more adaptive feature fusion calculation, which can lead to a richer and more accurate feature expression.

### 3.3. Spatial Stream

Generally speaking, for semantic segmentation tasks, the depth of the segmentation network [17,24] is very large, because only in this way can enough perception fields be obtained. Specifically, these networks mostly encode the input images by continuous down-sampling and convolution of the input, which results in rich semantic information and somewhat good predictions. However, in this process, the resolution of the output will be compressed many times, and thus the predicting boundary details still need to be improved. We can see from related work [17,31,38] that the maintenance of spatial information does have an impact on prediction accuracy.

**Figure 4:**
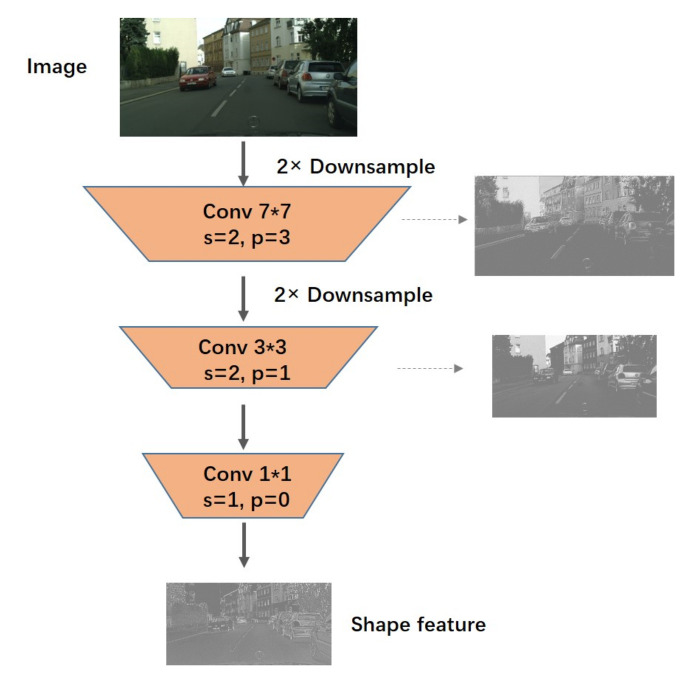
Structure diagram of spatial flow; the right-hand side is the visualization of the feature map after convolution of each layer. After three convolution layers, a quarter-size feature map containing spatial relations is obtained, as shown at the bottom of the figure.

Considering the importance of spatial information, based on the deep advanced semantic information extraction network, we also designed a shallow spatial flow to supplement the spatial information lost due to down-sampling in the deep path.The specific structure of the spatial flow branch is shown in Figure 4. It contains only three convolution layers, which are very simple. The first two layers both use a convolution layer with a stride of 2. The first and second convolution kernels are 7×7 and 3×3, respectively. Simple spatial information is extracted on the convolution kernels of different scales. The last convolution layer no longer reduces the feature size. The convolution layer of the 1×1 kernel is used to change the number of channels of the spatial flow for flexible adjustment of the amount of whole spatial information. The whole spatial flow only reduces the input to a quarter of the size, and the network structure is shallow. This design retains most of the spatial relations based on extracting the line color information, which is exactly what we need. Figure 4 shows the structure diagram of the spatial stream. The input image receives a quarter of the feature map containing spatial information through the spatial flow. In this process, we visualize the features of each layer. It can be seen that with the increase of convolution layers, rich spatial information is extracted.

### 3.4. Loss Function

The loss function used in our experiments is Online Hard Example Mining (OHEM) loss [39]. The core idea of this algorithm is to filter the input samples according to the loss of input samples. It filters out hard examples, which indicate the samples that have a great influence on classification, and then applies the filtered samples to training in Stochastic Gradient Descent (SGD) [40].

We treat the input picture as a pixel point sequence [$x_{1}$, $x_{2}$, $x_{3}$,..., $x_{N}$], where N is the number of pixels. For the pixel point Xi (i belongs to 1 to N), we can use Equation (5) to calculate the cross-entropy $CE(x_{i})$ of the point. $p_{x_{i}}$ is the probability that pixel $x_{i}$ is predicted to be the correct category

(5) $$CE\left(x_{i}\right)=-log\left(p_{x_{i}}\right).$$

The corresponding loss function expression is Equation (6) following the loss in BiseNet [41]:

(6) $$loss=-\frac{1}{N}\sum_{i=1}^{N}\sum_{j=1}^{C}y_{ij}log\left(p_{ij}\right).$$

where *C* is the number of categories, and $y_{ij}$ is a one-hot vector containing only 0 and 1 elements. If the category is the same as the category of the sample, take 1; otherwise, take 0. As for $p_{ij}$, it indicates the probability that the $i_{th}$ predicted sample belongs to category *j*.

For OHEM loss, entropy values are calculated from the input image pixel point sequence [$x_{1}$, $x_{2}$, $x_{3}$,..., $x_{N}$] according to Equation (5). Then, the sequence of new pixel points [$x^{\prime }$${}_{1}$, $x^{\prime }$${}_{2}$, $x^{\prime }$${}_{3}$, $x^{\prime }$${}_{4}$,..., $x^{\prime }$${}_{N}$] is obtained by sorting the entropy values from the largest to the smallest. We remove the last quarter of the small loss pixel points and train the first three-quarters of the larger loss targets. The corresponding OHEM loss function calculation formula is shown as Equation (7):

(7) $$loss_{ohem}=-\frac{1}{3N/4}\sum_{i=1}^{3N/4}\sum_{j=1}^{C}y_{ij}^{\prime }log\left(p_{ij}^{\prime }\right).$$

where $y_{ij}^{\prime }$ and $p_{ij}^{\prime }$ are the one-hot vectors after the pixel points are reordered and the probability of the predicted *j* class, respectively.

Drawing on what has been done before [13,37,41], we also use the auxiliary loss function in the network training. We designed a bypass output branch that consists of two convolution layers, namely a 3×3 convolution followed by a 1×1 convolution. The first convolution layer reduces the number of channels from 256 to 64, and the second layer directly reduces the number of channels to the number of label categories. Both convolution layers have a stride of 1, so the size of the stage one feature is not changed. To supervise this coarse segment prediction with the ground truth, we also need to sample the features four times to obtain the final rough segment result map we need.

Therefore, our loss function consists of two parts; one is the loss function $l_{out}$ calculated by the network output, and the other is the auxiliary loss function $l_{aux}$ of the coarse output branch. To optimize the loss function better, we give the auxiliary loss function a weight following PSPNet [17], which is expressed as follows:

(8) $$loss=l_{out}+\lambda \times l_{aux}.$$

Using such a joint loss function with an auxiliary loss function to supervise network learning will make our network easier to optimize.

## 4. Experiment

To verify the effectiveness of our proposed module, we have carried out a number of experiments on the Cityscapes dataset [19]. Our network achieved a competitive performance and has greatly improved on the baseline network. Additionally, we performed some visual contrast experiments to prove the effectiveness of our module.

The Cityscapes dataset, jointly provided by three German units including Daimler, contains stereo vision data of more than 50 cities for urban scene understanding, including 19 classes for urban scene analysis and pixel-level segmentation. It contains 2975 fine labeled images for training, 500 for validation, and 1525 for testing. Additionally, there are an additional 20,000 coarse segmentation labeled images for training. It is worth noting that all our experiments were conducted on the Cityscapes finely annotated set.

### 4.1. Implementation Details

Our network is based on PyTorch; following the setting of learning rate in previous work [13,16,17], we adopted the poly learning rate policy, where the learning rate of the current iteration can be calculated by way of multiplying by the factor (1 $-\frac{iter}{maxiter}$)${}^{0.9}$. We used a Stochastic Gradient Descent (SGD) optimizer [40] to optimize the network parameters. For the Cityscapes dataset, we set the initial learning rate of the network to 0.01, the weight decay coefficient to 0.0005, and the momentum to 0.9. In the network training process, we set the learning rate of the coarse segmentation output branch and feature balanced fusion module parts to 10 times, and the remaining parts to one time. The loss function is shown in Equation (8), and λ was set to 0.4 to achieve the best fusion effect. The OHEM [39] loss function was used as a category of network loss functions to purposefully improve the learning of difficult samples. In training, we replaced all BatchNorm layers with InPlaceABN-Sync [42]. For the data augmentation, the input pictures were randomly cropped to 876 × 876 sizes during the training and flipped horizontally. All experiments were performed on two Nvidia GTX 1080Ti GPUs. The total number of training iterations was set to 81k, and the first 1k iterations were warmup processes.

### 4.2. Experimental Results

Applying the methods we proposed and some common training techniques, and following the implementation rules described in Section 4.1, after training only on a finely annotated training set, our mIoU on the Cityscapes validation set reached 81.2% in terms of mIoU. Compared to the basic network DeepLabV3plus [2], we thus achieved an improvement of nearly 1.3%. Table 1 shows in detail the improvements we have achieved with other advanced networks in each class. Comparing with the IoUs class, it is not difficult to find that in most classes, our indicators have been greatly improved.

### 4.3. Ablation Study

In this section, we outline a series of performed comparative experiments to demonstrate the validity of our proposed modules.

#### 4.3.1. Baseline Network

Specifically, we set up two baseline networks as the basis of our experiments; one is ResNet101 [27], which is a very basic feature extraction network, and the other is ResNet101 designed with ASPP added to the end. We have made several experimental comparisons based on these two baselines.

**Baseline1: ResNet101-Dilated.** To make ResNet101 more suitable for semantic segmentation tasks, we set the dilation of the last two layers of ResNet as [1,2] and stride as [2,1]. Thus, the output stride of the network was set to 16, so that the feature mapping of 1/16 the image size was finally obtained. We pre-trained the network on the ImageNet dataset, and then fine-tuned the parameters on the Cityscapes dataset. After pre-training on the ImageNet dataset, subsequent training for specific segmented datasets converged faster.

**Baseline2: ResNet101+ASPP.** Just as we detailed in Section 2.2, ASPP is a module that has achieved tremendous success in semantics segmentation tasks due to its delicate design. To verify that our proposed module can also work with other modules to improve network performance, we also set up this ResNet101 + ASPP baseline. Its specific structure is composed of a global average pooling and three 3 × 3 dilated convolutions (dilated rates of 6, 12, and 18), which extract context information at multiple scales. Features from four branches are concatenated. Then, a 1 × 1 convolution was used to reduce the dimension of the concatenated map channel by 256.

**Inter-Level Feature Balanced Fusion Network.** The structural design of inter-level feature balanced fusion network is introduced in detail in Section 3.2. To make the best use of this module where appropriate, we have applied our designed inter-level feature balanced fusion module to each feature fusion process at two different levels in the network. Since we use the global average pooling operation to compute the inter-layer weights, this part of the calculation is not large (relative to [34,35,37]). We use inter-level feature balanced fusion module to balance the differences between two levels of features from ASPP and stage one’s low-level feature of the backbone network. Another time, inter-level feature balanced fusion strategy is applied in feature mapping fusion between a specially designed spatial branch and a previous backbone network.

**Inter-Level Feature Balanced Fusion Network with Spatial Stream.** The spatial stream was designed to be relatively simple so as to obtain more spatial information in the input image, and thus the spatial stream consists of only three layers of convolution, with the number of intermediate feature map channels mimicking the first two layers of the ResNet network structure. We set the number of intermediate channels to 64. However, because we were not sure how to control the amount of spatial information, we performed a set of comparative experiments. We set the number of spatial flow branches with different output channels to obtain some spatial information of different sizes. To obtain the optimal result, we finally set the number of spatial stream output channels to 128.

#### 4.3.2. Ablation Study for Inter-Level Feature Balanced Fusion Module

First, we used Baseline1 (101 and 50 of the atrous ResNet series) as the baseline network for the next series of experiments, and the corresponding output was directly up-sampled. The results of the experiment are shown in Table 2. We show the comparison data of two different depth backbone networks. To improve the network performance, we added the ASPP module at the end of the network, which improved the ResNet50 series by 2.80% and the ResNet101 series by 2.46% compared with the baseline network.

To verify the effectiveness of our proposed inter-level feature balance fusion module, we added layer skip-connections to the baseline network ResNet + ASPP. One of the fusion methods of skip-connection is the common concatenation corresponding to the “Skip-Connection” in Table 2, and the other is our feature balance fusion mode. From the experimental data in Table 2, it can be seen that adding layer skip-connection to ResNet50 and ResNet101 improved the performance respectively by 2.06% and 2.40%. The inter-level feature balanced fusion method has improved separately from the normal fusion method by 2.43% and 1.19% on ResNet50 and ResNet101, respectively.

#### 4.3.3. Ablation Study for Spatial Stream

In this section, we further analyze the importance of a spatial information stream to enhance experimental results. In Table 2, we find that adding spatial streams improved both ResNet50 and ResNet101, respectively by 0.34% and 0.11%. To obtain the most appropriate spatial flow design, we adjusted the spatial information of the spatial flow, corresponding to a series of convolution final output channels (16, 32, 48, 64, 128, 256). We controlled the same settings except for the number of channels. Six sets of comparative tests were performed on the Cityscapes validation set. As shown in Table 3, we found that when the number of final output channels is 128, the best prediction results can be obtained.

#### 4.3.4. Ablation Study for Improvement Strategies

As in [16,35,37], we also used similar improvement strategies, as shown in Table 4. (1) OHEM (online hard example mining): we focused on the training of difficult samples during the training process, which is reflected in the loss function in Section 3.4. We classified the samples predicted to be the correct kind with a probability of less than the threshold of 0.7 as difficult samples. With OHEM the performance on the Cityscapes validation set was improved by 1.48%. (2) DA (data augmentation with random scaling): during the training process, we randomly reduced or increased the size of the input pictures by 0.5, 0.75, 1.0, 1.25, 1.5, 1.75, or 2.0 times, and this increased the experimental results by 1.07%. (3) MS (multi scales): we averaged the inferred predictions of six scale input images to obtain the final predicted output, which had the sizes of 0.5, 0.75, 1.0, 1.25, 1.5, and 1.75 times. The experimental results were improved with MS by 1.21%. The final performance was 81.2% mIoU, higher than the 79.9% of Deeplabv3plus on the Cityscapes validation set, and we improved by about 1.3%.

We added auxiliary loss supervising identical to the loss function in Equation (8). Due to the uncertainty of the auxiliary loss’s weight combined with the main loss, which can lead to a better result if it is set properly, we conducted a set of comparative experiments. On the premise of ensuring the consistency of other parameters, we adjusted the coefficient of auxiliary loss from 0.1 to 1.0. The experimental results are shown in Table 5. After comparing the experimental data, we finally determined that when the weight parameter of auxiliary loss was set to 0.4, we could obtain the best performance on the Cityscapes validation set.

### 4.4. Visualization of Inter-Level Feature Balanced Fusion Module

**Figure 5:**
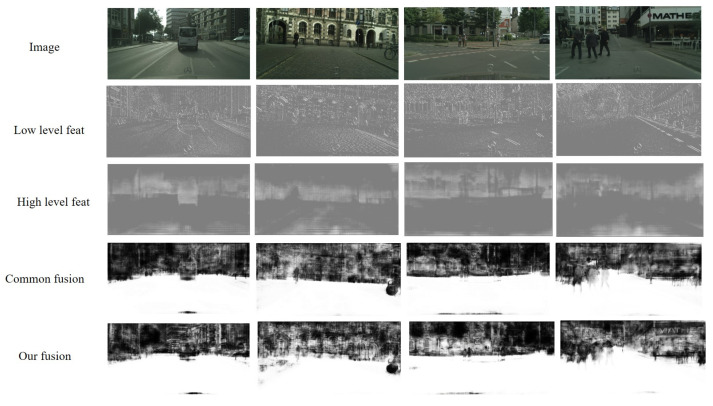
Visualization results of inter-level feature balanced fusion compared to common fusion on the Cityscapes set. For each line, we show an input image, and feature mapping from two levels. Meanwhile, we show the visualization of general fusion of two hierarchical features and visualization of fusion results using inter-level feature balanced fusion strategies.

In the previous part, we have introduced the network structure design and experimental data comparison. To show the role of our inter-level feature balanced fusion module in the network more vividly, we input an image to visualize the feature maps of different levels when the network processes this image. The visualization results are shown in Figure 5. We have visualized the low-level features of the network (in our case, the first stage of the backbone), which are the results of the second line in the figure. They mainly contain shallow spatial information and some line outlines. The third line is the deep feature, which contains rich semantic information, and the fourth line is the result of the fusion of two-level features obtained by the ordinary concatenation method. The result of inter-level feature balanced fusion is shown in the fourth line. The comparative results demonstrate that our fusion results can better integrate the two parts of features from different levels, and can carry clear line contour information while containing rich semantic information.

### 4.5. Comparing with the State-of-the-Art

On the Cityscapes test set, we further compared our proposed network with the recently published methods. We input the original test set images into the trained model to obtain the test set prediction results that meet the requirements of the official test set. In this reasoning process, we applied multi-scales prediction and flipping strategies, which should improve the performance of our network. We packaged and uploaded the results to the official test script, waited for more than ten minutes, and obtained the corresponding results, as shown in Table 6. Compared with other previous methods, our network achieved better performance. Compared with the replicated DeepLabV3plus [16], the mIoU index of our network on the Cityscapes test set exceeded it by 1.1%. In the training process, we only used the finely segmented data of the Cityscapes dataset to refine our network, which further demonstrates the effectiveness of the proposed method.

**Figure 6:**
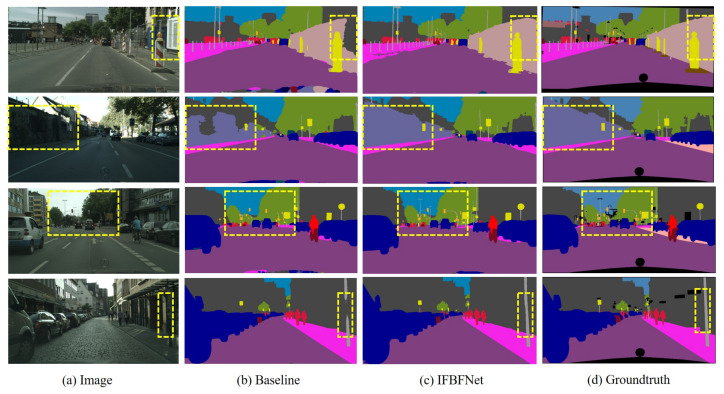
Visualization results of IFBFNet based on ResNet101 baseline on the Cityscapes validation set.

In Figure 6, we provide a comparison of predictions between our network and the baseline network. We visualize some samples of picture test results on the Cityscapes validation set for the baseline network and IFBFNet. We mark the areas where the results were significantly different with yellow lines. Looking at these graphs, we find that IFBFNet can significantly improve the prediction of object boundaries, and small objects, such as power poles, can also be well predicted to maintain the correct shape.

## 5. Conclusions

In this paper, IFBFNet is proposed for scene segmentation, which solves the problem that the information of feature fusion between semantic segmentation levels is mixed and unclear. Specifically, we adopt an adaptive feature fusion strategy to let the network learn by itself to reach a state where the low-level information and the high-level complex semantic information can be integrated more efficiently. At the same time, we add a shallow spatial information flow to increase the amount of spatial information. A series of ablation experiments and visualization of intermediate features have shown that using the inter-level feature balance fusion method can achieve a more balanced and clear feature representation, as well as a more accurate segmentation result by increasing the flow of spatial information. Our IFBFNet achieved very competitive results on the challenging Cityscapes dataset, significantly improving over baselines.

In the future, we will further explore more application scenarios of the inter-level feature balanced module. The inter-level feature balanced module we designed is mainly applied to the adaptive integration of different levels of information in the network structure. It can be extended to some scenarios that need to take into account both spatial information and high-level semantic information. The design we proposed is applied to the integration of two levels; considering the complex situation, such as the scene of feature fusion of three or more levels, one can refer to the same design, but we need to add corresponding input branches and weight blocks here. The effectiveness of this structure also needs to be verified in future work.

## Figures and Tables

**Table 1:** Comparison of our IFBFNet with DeepLabV3plus and other state-of-the-art networks on the Cityscapes validation set in terms of class IoUs and mean IoU.The blackened figure is the data with the highest index.

Methods	Road	s.Walk	Build	Wall	Fence	Pole	t-Light	t-Sign	Veg	Terrain	Sky	Person	Rider	Car	Truck	Bus	Train	Motor	Bike	Mean
LRR [43]	97.7	79.9	90.7	44.4	48.6	58.6	68.2	72.0	92.5	69.3	94.7	81.6	60.0	94.0	43.6	56.8	47.2	54.8	69.7	69.7
DeepLabV2 [25]	97.9	81.3	90.3	48.8	47.4	49.6	57.9	67.3	91.9	69.4	94.2	79.8	59.8	93.7	56.5	67.5	57.5	57.7	68.8	70.4
Piecewise [12]	98.0	82.6	90.6	44.0	50.7	51.1	65.0	71.7	92.0	**72.0**	94.1	81.5	61.1	94.3	61.1	65.1	53.8	61.6	70.6	71.6
PSPNet [17]	98.2	85.8	92.8	57.5	65.9	62.6	71.8	80.7	92.4	64.5	94.8	82.1	61.5	95.1	78.6	88.3	77.9	68.1	78.0	78.8
Multiscale [22]	-	-	-	-	-	-	-	-	-	-	-	-	-	-	-	-	-	-	-	77.8
DeeplabV3+ [2]	97.8	83.4	92.8	**67.6**	63.2	64.5	73.9	79.9	92.7	70.1	94.8	83.1	**67.4**	95.0	80.0	90.0	73.1	**71.6**	76.7	79.9
**Ours**	**98.2**	**86.0**	**93.9**	61.4	**67.5**	**66.8**	**74.8**	**81.4**	**93.2**	69.1	**95.4**	**84.7**	66.2	**95.7**	**87.0**	**90.1**	**84.2**	68.5	**78.2**	**81.2**

**Table 2:** Ablation experiments on ASPP, our inter-level feature balanced fusion module, and shape stream over the ResNet50 and ResNet101 base networks on the Cityscapes validation set. The blackened numbers represent the best performing group in this set of data.

BaseNet	ASPP	Skip-Connection	Feature Balanced Fusion	Spatial Stream	Mean IoU (%)
ResNet50					73.82
ResNet50	✓				76.62
ResNet50	✓	✓			78.68
ResNet50	✓	✓	✓		80.11
ResNet50	✓	✓	✓	✓	**80.45**
ResNet101					75.00
ResNet101	✓				77.46
ResNet101	✓	✓			79.86
ResNet101	✓	✓	✓		81.05
ResNet101	✓	✓	✓	✓	**81.16**

**Table 3:** Comparison of experimental data on the Cityscapes validation dataset of shape stream with different numbers of output channels.The blackened numbers represent the best performing group in this set of data.

**Channel**	16	32	48	64	128	256
**mIoU%**	80.75	80.13	80.99	80.90	**81.16**	80.70

**Table 4:** Performance comparison between different strategies on the Cityscape validation set. OHEM stands for online hard example mining loss function. DA represents data augmentation with random scaling. MS represents multi-scale inputs during inference.The blackened numbers represent the best performing group in this set of data.

Method	Backbone	OHEM	DA	MS	mIoU%
IFBFNet	ResNet101	×	×	×	77.40
IFBFNet	ResNet101	✓	×	×	78.88
IFBFNet	ResNet101	✓	✓	×	79.95
IFBFNet	ResNet101	✓	✓	✓	**81.16**

**Table 5:** Comparison of mIoU with different settings of the λ parameters in Equation (8), which control the size of the auxiliary loss.The blackened numbers represent the best performing group in this set of data.

**Methods**	λ=0.1	λ=0.2	λ=0.3	λ=0.4	λ=0.5	λ=0.6	λ=0.7	λ=0.8	λ=0.9	λ=1.0
**mIoU** (%)	80.29	81.03	80.99	**81.16**	80.51	80.72	81.03	79.97	79.97	80.64

**Table 6:** Per-class results on the Cityscapes testing set. Our network outperformed existing approaches and achieved 79.6% in MeanIoU.“-” indicates that the methods did not give the corresponding result.The blackened numbers represent the best performing group in this set of data.

Methods	Road	s.Walk	Build	Wall	Fence	Pole	t-Light	t-Sign	Veg	Terrain	Sky	Person	Rider	Car	Truck	Bus	Train	Motor	Bike	Mean
DeepLab-v2+CRF [25]	97.9	81.3	90.3	48.8	47.4	49.6	57.9	67.3	91.9	69.4	94.2	79.8	59.8	93.7	56.5	67.5	57.5	57.7	68.8	70.4
FRRN [3]	98.2	83.3	91.6	45.8	51.1	62.2	69.4	72.4	92.6	70	94.9	81.6	62.7	94.6	49.1	67.1	55.3	53.5	69.5	71.8
RefineNet [15]	98.2	83.3	91.3	47.8	50.4	56.1	66.9	71.3	92.3	70.3	94.8	80.9	63.3	94.5	64.6	76.1	64.3	62.2	70	73.6
DUC [44]	98.5	85.5	92.8	**58.6**	55.5	65	73.5	77.9	93.3	72	95.2	84.8	68.5	95.4	70.9	78.8	68.7	65.9	73.8	77.6
PSPNet [17]	-	-	-	-	-	-	-	-	-	-	-	-	-	-	-	-	-	-	-	78.4
ResNet-38 [45]	98.5	85.7	93.1	55.5	59.1	67.1	74.8	78.7	93.7	72.6	95.5	86.6	69.2	95.7	64.5	78.8	74.1	69	76.7	78.4
DeepLabV3+ [2]	98.6	86.3	92.9	**57.4**	59.5	64.6	73.0	77.6	93.4	72.5	95.5	85.6	69.5	95.7	69.0	**84.6**	74.6	67.2	75.0	78.5
**IFBFNet (ours)**	**98.7**	**87.0**	**93.3**	53.8	**60.7**	**67.7**	**76.4**	**80.1**	**93.8**	**73.7**	**95.7**	**87.3**	**72.3**	**96.2**	**70.7**	82.5	**75.4**	**70.2**	**77.4**	**79.6**

## Data Availability

Not applicable.
